# Laccase-Catalyzed Derivatization of Antibiotics with Sulfonamide or Sulfone Structures

**DOI:** 10.3390/microorganisms9112199

**Published:** 2021-10-21

**Authors:** Annett Mikolasch, Veronika Hahn

**Affiliations:** 1 Institute for Microbiology, University of Greifswald, Felix-Hausdorff-Str. 8, 17489 Greifswald, Germany; annett.mikolasch@uni-greifswald.de; 2 Interfaculty Institute for Genetics and Functional Genomics, University of Greifswald, Felix-Hausdorff-Str. 8, 17489 Greifswald, Germany; 3 Leibniz Institute for Plasma Science and Technology (INP), Felix-Hausdorff-Str. 2, 17489 Greifswald, Germany

**Keywords:** laccase, biotransformation, sulfonamide antibiotics, antibacterial activity, multidrug resistance (MDR), β-lactam antibiotics, methicillin-resistant *Staphylococcus aureus* (MRSA), antimicrobial resistance (AMR)

## Abstract

*Trametes* spec. laccase (EC 1.10.3.2.) mediates the oxidative coupling of antibiotics with sulfonamide or sulfone structures with 2,5-dihydroxybenzene derivatives to form new heterodimers and heterotrimers. These heteromolecular hybrid products are formed by nuclear amination of the *p*-hydroquinones with the primary amino group of the sulfonamide or sulfone antibiotics, and they inhibited in vitro the growth of *Staphylococcus* species, including multidrug-resistant strains.

## 1. Introduction

The evolution of antibiotic resistances amongst microorganisms is a global problem [1,2,3]. Because of this, novel antimicrobial substances and synthesis routes are needed. For this, enzymatic catalysis may be an alternative to conventional chemical synthesis processes [4,5]. The oxidoreductase laccase [E.C. 1.10.3.2, benzenediol:dioxygen oxidoreductase] possesses a number of advantages. The reactions can be performed under mild and environmentally friendly conditions, such as atmospheric pressure and room temperature. Additionally, no cofactors such as a coenzyme or NADPH are needed. 

Different laccase-mediated reactions are possible [6,7,8,9]. The laccase substrates may be oxidized by the enzyme and can then react to form homomolecular products [10,11], or the oxidized substrate may react with other compounds, such as amines or thiols, resulting in heteromolecular products [12]. These hybrid products are formed by C−C [13,14], C−N [15,16], C−O [17], or C−S [18,19] bonds. In addition, the reactions may also lead to multiple bond formation by ring closure mechanisms [20,21].

These diverse reaction types resulted in the synthesis or derivatization of antitumor [22,23,24], antioxidative [25,26,27], or estrogenic [28,29] compounds. Additionally, the synthesis of antibiotics by homomolecular and heteromolecular reactions have also been described. Thus, the laccase-catalyzed dimerizations of different *ortho*-aminophenol derivatives by C=N and C−O bond formations resulted in phenoxazinone pigments proposed as antitumor antibiotics [30,31,32,33,34,35]. Additionally, the catalytic derivatization of β-lactam antibiotics has been of particular interest. Agematu et al. [36] described the laccase-mediated synthesis of homomolecular dimers from different esters of penicillin X. The dimers of the penicillin X methyl ester showed hardly any antibacterial activity. In contrast, the *ortho*-*ortho* coupling product formed from penicillin X pivaloyloxymethyl ester represented a precursor (prodrug) of the penicillin X dimer. However, the minimum inhibitory concentration was lower than that of penicillin X sodium salt [36]. The laccase-catalyzed oxidation of a cephalosporanic acid resulted in the formation of two diastereomers, which differed in their configuration at C−2′ and their antibacterial efficacy [37]. Nevertheless, both compounds showed lower activity than the parent compound. Furthermore, penicillins and cephalosporines with free amino groups were derivatized with dihydroxylated aromatic compounds resulting in the formation of heteromolecular products [38,39,40]. The newly synthesized heterodimers had a moderate to high growth inhibitory effect against various bacteria including multidrug-resistant *Staphylococcus* and *Enterococcus* species. The products possessed also in vivo efficacy against *Staphyloccocus aureus* in mice. Additionally, the dimers resulting from reactions of 2,5-dihydroxybenzoic acid derivatives and penicillins or cephalosporins showed no cytotoxicity [41,42].

Consequently, 2,5-dihydroxybenzene derivatives were used for the derivatization of sulfanilamide and structurally related substances. Sulfanilamide is a structural part of 4′-sulfonamide-2,4-diaminoazobenzene (trade name: Prontosil), which was the first antibiotic of the sulfonamide group. This antibiotic was used against Gram-positive cocci such as *Streptococcus* spp. involved in the induction of sepsis [43]. Because of their structural similarity, sulfonamide acts as an antagonist for *para*-aminobenzoic acid. This results in the inhibition of bacterial folic acid synthesis, which is essential for prokaryotes [44,45]. The antibacterial effect of the sulfone dapsone relies on the same antagonistic mechanism [46]. The increasing incidence of antibiotic resistance shows the need for the synthesis of novel compounds. In the present study, the extracellular laccase C of *Trametes* spec. was employed for the production of heterodimers in reactions of sulfanilamide and its derivatives with *para*-dihydroxylated aromatic substances. The resulting products were tested for their antibacterial efficacy and cytotoxicity to human cells.

Different *Staphylococcus* strains were chosen for the determination of the antibacterial efficacy of the products formed. *S. aureus* ATCC6538/DSM799 (American Type Culture Collection/German Collection of Microorganisms and Cell Cultures) and the patient-derived multidrug-resistant *S. epidermidis* 99847 were used. Additionally, the northern German epidemic MRSA (methicillin-resistant *S. aureus*; Norddeutscher Epidemiestamm), which is resistant to penicillin, oxacillin, gentamicin, erythromycin, clindamycin, oxytetracycline, trimethoprim-sulfamethoxazole, rifampicin, and ciprofloxacin was employed [47]. Cytotoxicity was tested in a neutral red uptake assay using FL cells (human amniotic epithelial cell line).

## 2. Materials and Methods

### 2.1. Enzyme

Extracellular laccase C of *Trametes* spec. (EC 1.10.3.2) with an activity of 800 nmol·mL^−1^·min^−1^ (substrate: 2,2′-amino-bis-3-ethylbenzthiazoline-6-sulfonic acid) was obtained from ASA Spezialenzyme (Wolfenbüttel, Germany) and used in all experiments. 

### 2.2. Substrates and Conditions of Biotransformation

The antibiotics—sulfanilamide, sulfamerazine and dapsone (2 mM)—were dissolved in 60 mL sodium acetate buffer, 20 mM pH 5.6. After addition of laccase C, the 2,5-dihydroxybenzene derivatives—2,5-dihydroxy-*N*-(2-hydroxyethyl)benzamide, 2,5-dihydroxybenzoic acid methyl ester, 2,5-dihydroxyacetophenone, 2,5-dihydroxyphenylacetic acid, or 2,5-dihydroxy-1,4-benzenediacetic acid—were added (6 mL of a 20 mM solution in sodium acetate buffer, pH 5.6). The reaction mixtures were incubated for 6 h at room temperature (RT) with agitation at 400 rpm.

Chemicals were purchased from the commercial supplier Sigma-Aldrich (Steinheim, Germany) with the exception of 2,5-dihydroxy-*N*-(2-hydroxyethyl)benzamide purchased from Midori Kagaku Co (Tokyo, Japan).

### 2.3. Isolation of Biotransformation Products

The isolation of the coupling products was performed by solid phase extraction described in detail by Mikolasch et al. [38].

### 2.4. Analytical High-Performance Liquid Chromatography (HPLC)

Samples of the incubation mixture were analyzed by HPLC−UV/Vis detector for routine analyses [38].

### 2.5. Characterization of Biotransformation Products

Products were analyzed by mass spectrometry (LC/MS with API-ES in negative and positive modes). The nuclear magnetic resonance (NMR) spectra were obtained at 300 MHz (^1^H) in acetonitrile-*d_3_*. Chemical shifts (δ) are given in part per million (ppm) downfield relative to tetramethylsilane (TMS, SiMe_4_), and spin-spin coupling constants (*J*) are in Hz. Residual solvent central signal was recorded at δ_H_ = 1.93 ppm and δ_C_ = 1.3 ppm.

**3,6-Dioxo-2-(4-sulfamoylanilino)cyclohexa-1,4-diene-1-carboxyl methyl ester 3a_1_.** Yield 88%, ^1^H NMR (300 MHz, CH_3_CN-*d*_3_): δ (ppm) 3.11 (s, 3H, H−8), 5.77 (s, 2H, H−8′), 6.70 (d, ^3^*J* = 10.15 Hz, 1H, H−5), 6.81 (d, ^3^*J* = 10.15 Hz, 1H, H−4), 7.25 (d, ^3^*J* = 8.75 Hz, 2H, H−2′, H−6′), 7.79 (d, ^3^*J* = 8.75 Hz, 2H, H−3′, H−5′), 13.41 (s, 1H, H−7′), ^13^C NMR (75 MHz, CH_3_CN-*d*_3_): δ 51.6 (C−8), 102.4 (C−1), 125.6 (C−2′, C−6′), 128.3 (C−3′, C−5′), 133.1 (C−5), 139.7 (C−4′), 141.1 (C−4), 145.5 (C−1′), 154.5 (C−2), 169.2 (C−7), 184.5 (C−3), 185.4 (C−6) ppm. LC/MS *m*/*z* 337.1 [M + H]^+^ API-ES pos. mode, 335.1 [M − H]^−^ API-ES neg. mode.

**N-(2-Hydroxyethyl)-3,6-dioxo-2-(4-sulfamoylanilino)cyclohexa-1,4-diene-1-carboxamide 3b_1_.** Yield 92%, ^1^H NMR (300 MHz, CH_3_CN-*d*_3_): δ (ppm) 3.40 (m, ^3^*J* = 5.4 Hz, 2H, H−9), 3.59 (t, ^3^*J* = 5.4 Hz, 2H, H−10), 5.74 (s, 2H, H−8′), 6.63 (d, ^3^*J* = 10.25 Hz, 1H, H−5), 6.71 (d, ^3^*J* = 10.25 Hz, 1H, H−4), 7.26 (d, ^3^*J* = 8.7 Hz, 2H, H−2′, H−6′), 7.79 (d, ^3^*J* = 8.8 Hz, 2H, H−3′, H−5′), 9.49 (s(broad), 1H, H−8), 13.42 (s, 1H, H−7′), ^13^C NMR (75 MHz, CH_3_CN-*d*_3_): δ 42.3 (C−9), 61.4 (C−10), 101.4 (C−1), 125.2 (C−2′, C−6′), 128.1 (C−3′, C−5′), 133.3 (C−5), 139.9 (C−4′), 141.4 (C−4), 145.3 (C−1′), 153.5 (C−2), 170.2 (C−7), 184.6 (C−3), 185.5 (C−6) ppm. LC/MS *m*/*z* 366.1 [M + H]^+^ API-ES pos. mode, 364.1 [M − H]^−^ API-ES neg. mode.

**4-(2-Acetyl-3,6-dihydroxy-anilino)benzenesulfonamide 3c_2_.** Yield 10% (only available in a mixture with trimers), LC/MS *m*/*z* 323.1 [M + H]^+^, 345.1 [M + Na]^+^ API-ES pos. mode.

**3,6-Dihydroxy-2-[4-[(4-methylpyrimidin-2-yl)sulfamoyl]anilino]benzoic acid methyl ester 3d.** Yield 81%, ^1^H NMR (300 MHz, CH_3_CN-*d*_3_): δ (ppm) 2.32 (s, 3H, H−13′), 3.10 (s, 3H, H−8), 3.15 (s, 1H, H−8′), 6.65 (d, ^3^*J* = 8.9 Hz, 1H, H−5), 6.76 (d, ^3^*J* = 8.9 Hz, 1H, H−4), 6.86 (d, ^3^*J* = 5.1 Hz, 1H, H−11′), 7.25 (d, ^3^*J* = 8.7 Hz, 2H, H−2′, H−6′), 7.95 (d, ^3^*J* = 8.7 Hz, 2H, H−3′, H−5′), 8.27 (d, ^3^*J* = 5.1 Hz, 1H, H−12′), 13.41 (s, 1H, H−7′), ^13^C NMR (75 MHz, CH_3_CN-*d*_3_): δ 22.6 (C−13′), 51.7 (C−8), 114.5 (C−2′, C−6′), 115.1 (C−5), 118.3 (C−4), 120.2 (C−1), 120.4 (C−11′), 124.3 (C−2), 128.9 (C−3′, C−5′), 130.7 (C−4′), 146.1 (C−3), 148.3 (C−10′), 150.4 (C−6), 152.2 (C−1′), 156.3 (C−12′), 163.7 (C−9′), 167.4 (C−7) ppm. ^1^H and ^13^C NMR data resulted in hydroquinoid structure **3d_2_**. LC/MS *m*/*z* 427.3 [M − H]^−^ API-ES neg. mode, 855 [2M − H]^−^ API-ES neg. mode, LC/MS data resulted in quinoid structure **3d_1_**.

**3,6-Dihydroxy-*N*-(2-hydroxyethyl)-2-[4-[(4-methylpyrimidin-2-yl)sulfamoyl]anilino]benzamide 3e.** Yield 93%, ^1^H NMR (300 MHz, CH_3_CN-*d*_3_): δ (ppm) 2.32 (s, 3H, H−13′), 3.15 (s, 1H, H−8′), 3.35 (m, ^3^*J* = 5.5 Hz, 2H, H−9), 3.57 (t, ^3^*J* = 5.5 Hz, 2H, H−10), 6.63 (d, ^3^*J* = 8.9 Hz, 1H, H−5), 6.74 (d, ^3^*J* = 8.9 Hz, 1H, H−4), 6.86 (d, ^3^*J* = 5.1 Hz, 1H, H−11′), 7.23 (d, ^3^*J* = 8.7 Hz, 2H, H−2′, H−6′), 7.95 (d, ^3^*J* = 8.7 Hz, 2H, H−3′, H−5′), 8.27 (d, ^3^*J* = 5.1 Hz, 1H, H−12′), 9.45 (s, 1H, H−8), 13.30 (s, 1H, H−7′), ^13^C NMR (75 MHz, CH_3_CN-*d*_3_): δ 22.7 (C−13′), 42.1 (C−9), 61.2 (C−10), 114.3 (C−2′, C−6′), 115.5 (C−5), 118.6 (C−4), 120.5 (C−1), 120.7 (C−11′), 124.8 (C−2), 128.9 (C−3′, C−5′), 130.8 (C−4′), 146.2 (C−3), 148.2 (C−10′), 150.2 (C−6), 152.2 (C−1′), 156.1 (C−12′), 163.8 (C−9′), 167.3 (C−7) ppm. ^1^H and ^13^C NMR data resulted in hydroquinoid structure **3e_2_**. LC/MS *m*/*z* 456.3 [M − H]^−^ API-ES neg. mode, 914 [2M − H]^−^ API-ES neg. mode, LC/MS data resulted in quinoid structure **3e_1_**.

**4-[(2-Acetyl-3,6-dioxo-cyclohexa-1,4-dien-1-yl)amino]-*N*-(4-methylpyrimidin-2-yl)benzenesulfonamide 3f_1_ and 4-(2-acetyl-3,6-dihydroxy-anilino)-*N*-(4-methylpyrimidin-2-yl)benzenesulfonamide 3f_2_.** No yields of isolated products (only available in a mixture of dimers and trimers), LC/MS *m*/*z* 411.1 [M − H]^−^ API-ES neg. mode, 823 [2M − H]^−^ API-ES neg. mode, LC/MS *m*/*z* 413.1 [M − H]^−^ API-ES neg. mode, 827 [2M − H]^−^ API-ES neg. mode, LC/MS data resulted in quinoid and hydroquinoid structures.

**4-[[5-Acetyl-3,6-dioxo-4-(4-sulfamoylanilino)cyclohexa-1,4-dien-1-yl]amino]benzene-sulfonamide 4c_1_.** Yield 80%, ^1^H NMR (300 MHz, CH_3_CN-*d*_3_): δ (ppm) 2.50 (s, 3H, H−8), 5.74 (s, 2H, H−8′), 5.77 (s, 2H, H−8′), 6.09 (s, 1H, H−4), 7.28 (d, ^3^*J* = 8.6 Hz, 2H, H−2′, H−6′), 7.48 (d, ^3^*J* = 8.6 Hz, 2H, H−2′, H−6′), 7.80 (d, ^3^*J* = 8.6 Hz, 2H, H−3′, H−5′), 7.87 (d, ^3^*J* = 8.6 Hz, 2H, H−3′, H−5′), 13.30 (s, 1H, H−7′), 13.42 (s, 1H, H−7′). LC/MS *m*/*z* 491.1 [M + H]^+^ API-ES pos. mode.

**4-[[5-Acetyl-4-[4-[(4-methylpyrimidin-2-yl)sulfamoyl]anilino]-3,6-dioxo-cyclohexa-1,4-dien-1-yl]amino]-*N*-(4-methylpyrimidin-2-yl)benzenesulfonamide 4f_1_ and 4-[3-acetyl-2,5-dihydroxy-4-[4-[(4-methylpyrimidin-2-yl)sulfamoyl]anilino]anilino]-*N*-(4-methylpyrimidin-2-yl)benzenesulfonamide 4f_2_.** No yields of isolated products (only available in a mixture of dimers and trimers), LC/MS *m*/*z* 673.4 [M − H]^−^ API-ES neg. mode, LC/MS *m*/*z* 675.4 [M − H]^−^ API-ES neg. mode, LC/MS data resulted in quinoid and hydroquinoid structure.

**2-[3,6-Dihydroxy-2-(4-sulfamoylanilino)phenyl]acetic acid 5a**. Yield 48%, ^1^H NMR (300 MHz, CH_3_CN-*d*_3_): δ (ppm) 3.47 (s, 2H, H−7), 5.75 (s, 2H, H−8′), 6.78 (d, ^3^*J* = 8.6, 1H, H−5), 6.80 (d, ^3^*J* = 8.6, 1H, H−4), 7.53 (d, ^3^*J* = 8.7 Hz, 2H, H−2′, H−6′), 7.84 (d, ^3^*J* = 8.7 Hz, 2H, H−3′, H−5′), 13.41 (s, 1H, H−7′), ^1^H NMR data resulted in hydroquinoid structure **5a_2_**. LC/MS *m*/*z* 335.1 [M − H]^−^ API-ES neg. mode, 671 [2M−H]^−^ API-ES neg. mode, LC/MS data resulted in quinoid structure **5a_1_**.

**2-[4-(Carboxymethyl)-3,6-dihydroxy-2-(4-sulfamoylanilino)phenyl]acetic acid 5b**. Yield 35%, ^1^H NMR (300 MHz, CH_3_CN-*d*_3_): δ (ppm) 3.44 (s, 2H, H−7), 3.51 (s, 2H, H−7), 5.77 (s, 2H, H−8′), 6.42 (s, 1H, H−5), 7.52 (d, ^3^*J* = 8.7 Hz, 2H, H−2′, H−6′), 7.81 (d, ^3^*J* = 8.7 Hz, 2H, H−3′, H−5′), 13.40 (s, 1H, H−7′), ^1^H NMR data resulted in hydroquinoid structure **5b_2_**. LC/MS *m*/*z* 393.1 [M − H]^−^ API-ES neg. mode, 787 [2M−H]^−^ API-ES neg. mode, LC/MS data resulted in quinoid structure **5b_1_**.

**2-[2-[4-[(4-Methylpyrimidin-2-yl)sulfamoyl]anilino]-3,6-dioxo-cyclohexa-1,4-dien-1-yl]acetic acid 5c_1_ and 2-[3,6-dihydroxy-2-[4-[(4-methylpyrimidin-2-yl)sulfamoyl]anilino]phenyl]acetic acid 5c_2_.** No yields of isolated products. LC/MS *m*/*z* 427.1 [M − H]^−^ API-ES neg. mode, LC/MS *m*/*z* 429.1 [M − H]^−^ API-ES neg. mode, LC/MS data resulted in quinoid and hydroquinoid structure.

**2-[4-(Carboxymethyl)-5-[4-[(4-methylpyrimidin-2-yl)sulfamoyl]anilino]-3,6-dioxo-cyclohexa-1,4-dien-1-yl]acetic acid 5d_1_ and 2-[4-(carboxymethyl)-2,5-dihydroxy-3-[4-[(4-methylpyrimidin-2-yl)sulfamoyl]anilino]phenyl]acetic acid 5d_2_.** No yields of isolated products. LC/MS *m*/*z* 485.1 [M − H]^−^ API-ES neg. mode, LC/MS *m*/*z* 487.1 [M − H]^−^ API-ES neg. mode, LC/MS data resulted in quinoid and hydroquinoid structure.

**2-[4-[4-[(2-Methoxycarbonyl-3,6-dioxo-cyclohexa-1,4-dien-1-yl)amino]phenyl]sulfonylanilino]-3,6-dioxo-cyclohexa-1,4-diene-1-carboxyl methyl ester 6a_1_.** LC/MS *m*/*z* 577.1 [M + H]^+^ API-ES pos. mode, LC/MS data resulted in a quinoid structure.

**N-(2-Hydroxyethyl)-2-[4-[4-[[2-(2-hydroxyethylcarbamoyl)-3,6-dioxo-cyclohexa-1,4-dien-1-yl]amino]phenyl]sulfonylanilino]-3,6-dioxo-cyclohexa-1,4-diene-1-carboxamide 6b_1_**. LC/MS *m*/*z* 635.1 [M + H]^+^ API-ES pos. mode, LC/MS data resulted in a quinoid structure.

**3,6-Dihydroxy-2-[4-[4-[(2-methoxycarbonyl-3,6-dioxo-cyclohexa-1,4-dien-1-yl)amino]phenyl]sulfonylanilino]benzoic acid methyl ester 6a_2_.** LC/MS *m*/*z* 579.1 [M + H]^+^ API-ES pos. mode, LC/MS data resulted in a quinoid-hydroquinoid structure.

**3,6-Dihydroxy-*N*-(2-hydroxyethyl)-2-[4-[4-[[2-(2-hydroxyethylcarbamoyl)-3,6-dioxo-cyclohexa-1,4-dien-1-yl]amino]phenyl]sulfonylanilino]benzamide 6b_2_.** LC/MS *m*/*z* 637.1 [M + H]^+^ API-ES pos. mode, LC/MS data resulted in a quinoid-hydroquinoid structure.

**2-[4-[4-(3,6-Dihydroxy-2-methoxycarbonyl-anilino)phenyl]sulfonylanilino]-3,6-dihydroxy-benzoic acid methyl ester 6a_3_**. Yield 22%, ^1^H NMR (300 MHz, CH_3_CN-*d*_3_): δ (ppm) 3.14 (s, 6H, H−8), 6.62 (d, ^3^*J* = 8.7 Hz, 2H, H−5), 6.78 (d, ^3^*J* = 8.7 Hz, 2H, H−4), 7.25 (d, ^3^*J* = 8.8 Hz, 4H, H−2′, H−6′), 7.85 (d, ^3^*J* = 8.8 Hz, 4H, H−3′, H−5′), 13.25 (s, 2H, H−7′). LC/MS *m*/*z* 581.1 [M + H]^+^ API-ES pos. mode, LC/MS and ^1^H NMR data resulted in a hydroquinoid structure.

**2-[4-[4-[3,6-dihydroxy-2-(2-hydroxyethylcarbamoyl)anilino]phenyl]sulfonylanilino]-3,6-dihydroxy-*N*-(2-hydroxyethyl)benzamide 6b_3_**. Yield 28%, ^1^H NMR (300 MHz, CH_3_CN-*d*_3_): δ (ppm) 3.25 (m, ^3^*J* = 5.4 Hz, 4H, H−9), 3.54 (t, ^3^*J* = 5.4 Hz, 4H, H−10), 6.61 (d, ^3^*J* = 8.7 Hz, 2H, H−5), 6.76 (d, ^3^*J* = 8.7 Hz, 2H, H−4), 7.28 (d, ^3^*J* = 8.8 Hz, 4H, H−2′, H−6′), 7.88 (d, ^3^*J* = 8.8 Hz, 4H, H−3′, H−5′), 9.45 (s, 2H, H−8), 13.25 (s, 2H, H−7′). LC/MS *m*/*z* 639.1 [M + H]^+^ API-ES pos. mode, LC/MS and ^1^H NMR data resulted in a hydroquinoid structure.

### 2.6. Determination of Antibacterial Activity

An agar diffusion method described by Mikolasch et al. [39] was used to determine the antibacterial activity in the range from 29 to 1490 nmol. In brief, sterile Mueller-Hinton II-Agar in petri dishes (Becton Dickinson Microbiology systems, Cockeysville, Maryland, U.S.A.) was inoculated with 200 µL of bacterial solution (bacterial cell suspension 15 × 10^7^ cells). The bacterial strains *Staphylococcus aureus* ATCC6538/DSM799, northern German epidemic MRSA (methicillin-resistant *S. aureus*; Norddeutscher Epidemiestamm), and the multidrug-resistant strain isolated from patients *S. epidermidis* 99847 were used. The test substances were applied on sterile paper discs (Sensi-Disc, 6 mm diameter, Becton Dickinson Microbiology systems). Petri dishes were kept for 3 h in a refrigerator (for prediffusion) and were then incubated for 24 h at 37 °C. Average inhibition zone diameters were calculated from 3 replicates.

### 2.7. Cytotoxic Activity

Cytotoxicity was determined by a neutral red uptake assay using FL-cells, a human amniotic epithelial cell line, as reported previously [39].

## 3. Results

### 3.1. Biotransformation of Sulfanilamide and Sulfamerazine with 2,5-Dihydroxybenzoic Acid Methyl Ester, 2,5-Dihydroxy-N-(2-Hydroxyethyl)Benzamide, or 2,5-Dihydroxyacetophenone by Laccase

**Figure 1:**
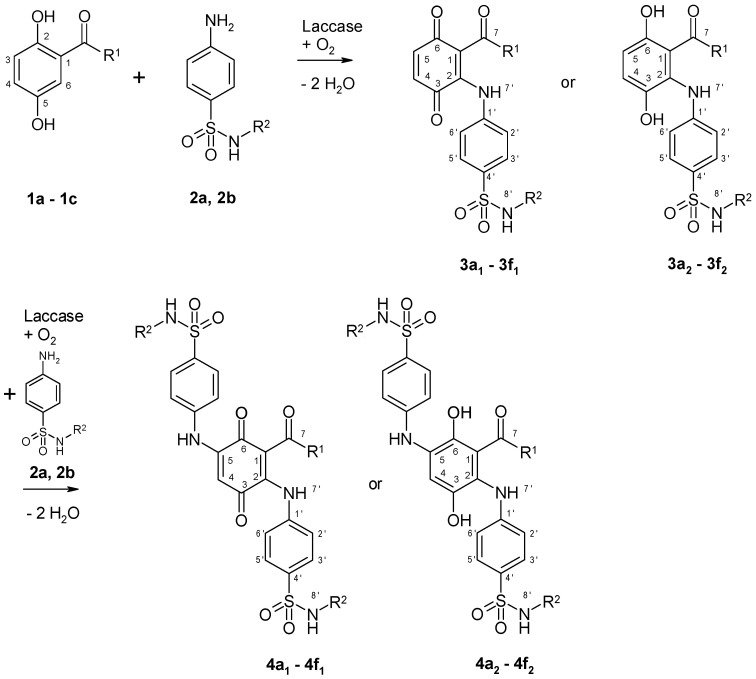
Laccase-mediated reactions of 2,5-dihydroxybenzene derivatives (**1a**–**1c**) with sulfonamides (**2a**, **2b**) for the synthesis of heterodimers and heterotrimers produced as shown in Table 1. (R^1^=OCH_3,_ NH(CH_2_)_2_OH, CH_3_; R^2^=H, CNC(CH_3_)(CH)_2_N).

Laccase-mediated reactions of 2,5-dihydroxybenzoic acid methyl ester (**1a**) with sulfanilamide (**2a**) resulted in a heteromolecular hybrid dimer with quinoid structure (**3a_1_**). In a similar way, the reaction of 2,5-dihydroxy-*N*-(2-hydroxyethyl)benzamide (**1b**) with sulfanilamide (**2a**) resulted in the production of structure **3b_1_** (Figure 1 and Table 1).

2,5-Dihydroxyacetophenone (**1c**) reacts with **2a** by laccase mediation to a heteromolecular hybrid dimer with the hydroquinoid structure **3c_2_**, and to a heteromolecular hybrid trimer with quinoid structure **4c_1_**. When sulfamerazine (**2b**) was used as reaction partner, the product pattern changed. The sulfonamide **2b** reacted with **1a** to form heteromolecular hybrid dimers, which showed hydroquinoid character (**3d_2_**) in NMR measurements and quinoid character (**3d_1_**) during LC/MS analyses. The same results were obtained for the laccase-mediated reaction of **1b** with sulfamerazine (**3e_1_**, **3e_2_**), whereas **1c** reacted with this sulfonamide to yield a mixture of dimers (**3f_1_**, **3f_2_**) and trimers (**4f_1_**, **4f_2_**).

After separation of **3a_1_** and **3b_1_** by solid phase extraction, LC/MS analyses in negative and positive mode showed molecular masses of these products attributed to the formation of heteromolecular hybrid dimers each consisting of a structural part of a 2,5-dihydroxybenzene derivative (**1a** or **1b**) coupled to **2a** accompanied by the loss of four hydrogen atoms. These couplings were confirmed by the presence of characteristic signals for **1a** or **1b** and for **2a** in the ^1^H NMR spectra of **3a_1_** and **3b_1_** as well as by the presence of all carbons of **1a** or **1b** and of **2a** in the ^13^C−NMR spectra of the products, but two signals in the range of 180 ppm indicated a quinoid character of these products. The number of CH proton signals of **1a** and **1b** changed from three in the substrate, to two signals in the products. The multiplicity of the proton signals H−4 and H−5 indicated an additional substituent at the C−2 atom and the loss of a proton. The chemical shift to lower field of the H−4 and H−5 signals confirmed the presence of an electron-withdrawing group. Signals for phenolic hydroxyl groups could not be measured, but instead additional amine protons were detected. All analytical data confirmed the oxidation of the *p*-hydroquinone structure of **1a** and **1b** to a quinone. The heteromolecular hybrid dimers **3a_1_** and **3b_1_** were formed by nuclear amination of the *p*-hydroquinones **1a** or **1b** at the C−6 position with the primary amino group of **2a** (Figure 1). 

Educt **1c** reacted with **2a** to a heteromolecular hybrid dimer **3c_2_**, which was only analyzed by LC/MS in positive mode in a mixture together with a heteromolecular hybrid trimer **4c_1_**. From the LC/MS data of **3c_2_** we deduced a dimer with hydroquinoid structure. The product **4c_1_** was described by LC/MS and ^1^H NMR analyses. All data showed a heteromolecular hybrid trimer consisting of a structural part of **1c** coupled to two molecules of **2a** accompanied by the loss of six hydrogen atoms. The number of CH proton signals of **1c** changed from three in the substrate, to one singlet in the product. The data of this proton signal H−4 indicated an additional substituent at the C−2 atom and a second substituent at the C−5 atom of the product. The chemical shift of the H−4 signal verified the presence of electron-withdrawing groups. Furthermore, the two amine protons detected confirmed the coupling of two molecules of **2a** to one molecule of **1c** accompanied by the formation of a heteromolecular hybrid trimer with quinoid structure **4c_1_**.

Like the reactions of **1a** or **1b** with **2a**, the reactions of **1a** or **1b** with sulfamerazine (**2b**) also showed the same reaction patterns. Whereas **1a** or **1b** each reacted with **2a** to yield a heteromolecular hybrid dimer with clear quinoid structure (**3a_1_** and **3b_1_**), the reaction of **1a** or **1b** with **2b** each resulted in a heteromolecular hybrid dimer, which yielded the hydroquinoid structures **3d_2_** and **3e_2_** by ^1^H NMR and ^13^C NMR, and as the quinoids **3d_1_** and **3e_1_** by LC/MS.

From the reaction of **1c** with **2b** only a product mixture could be isolated and measured by LC/MS. Data for heteromolecular hybrid dimers with quinoid **3f_1_** and hydroquinoid **3f_2_** structure and for heteromolecular hybrid trimers with quinoid **4f_1_** and hydroquinoid **4f_2_** structure were detected. In this case, pure substances could not be isolated.

### 3.2. Biotransformation of Sulfanilamide and Sulfamerazine with 2,5-Dihydroxyphenylacetic Acid or 2,5-Dihydroxy-1,4-Benzenediacetic Acid by Laccase

**Figure 2:**
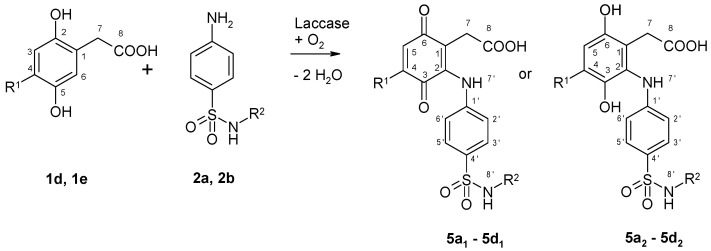
Laccase-mediated reactions of 2,5-dihydroxyphenylacetic acid (**1d**) and 2,5-dihydroxy-1,4-benzenediacetic acid (**1e**) with sulfonamides (**2a**, **2b**) for the synthesis of heterodimers produced as shown in Table 2. (R^1^=H, CH_2_COOH; R^2^=H, CNC(CH_3_)(CH)_2_N).

2,5-Dihydroxyphenylacetic acid (**1d**) or 2,5-dihydroxy-1,4-benzenediacetic acid (**1e**) coupled with sulfanilamide (**2a**) proceeded quite differently from **1a** and **1b** (Figure 2 and Table 2). Whereas **1a** or **1b** each reacted with **2a** to yield a heteromolecular hybrid dimer with clear quinoid structure (**3a_1_** and **3b_1_**), the reaction of **1d** or **1e** with **2a** each resulted in a heteromolecular hybrid dimer, which were determined to be the hydroquinoids **5a_2_** and **5b_2_** by ^1^H NMR and the quinoids **5a_1_** and **5b_1_** by LC/MS.

In contrast, the product formation of **1d** or **1e** with sulfamerazine (**2b**) was comparable to that of **1a** or **1b**. Thus, the respective heteromolecular hybrid dimers with quinoid and hydroquinoid structures were detected in all four reactions. The only difference is that **1d** or **1e** reacted with **2b** only to a product mixture from which no pure substance could be isolated and only LC/MS measurements were possible whereas from the reaction of **1a** or **1b** with **2b** products were isolated and analyzed both by LC/MS and by NMR. But all of these four reactions are expected to follow the same reaction mechanism. In general, the reactions of **1d** or **1e** with **2a** or **2b** did not lead to isolated pure substances whereas the reactions with **1a** or **1b** resulted in products, which could be isolated.

### 3.3. Biotransformation of Dapsone with 2,5-Dihydroxybenzoic Acid Methyl Ester or 2,5-Dihydroxy-N-(2-Hydroxyethyl)Benzamide by Laccase

**Figure 3:**
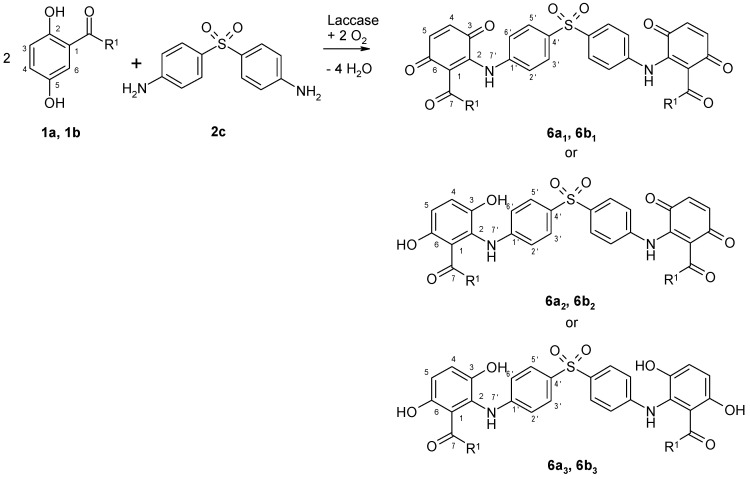
Laccase-mediated reactions of 2,5-dihydroxybenzene derivatives (**1a, 1b**) with dapsone (**2c**) for the synthesis of heterotrimers produced as shown in Table 3. (R^1^=OCH_3,_ NH(CH_2_)_2_OH).

The 2,5-dihydroxybenzoic acid methyl ester (**1a**) or 2,5-dihydroxy-*N*-(2-hydroxyethyl)benzamide (**1b**) reacted with dapsone (**2c**) as amino reaction partner by Michael addition resulting in heteromolecular products, which were detected by characteristic UV-Vis spectra on HPLC for both substances (Figure 3 and Table 3).

After purification of the products by solid phase extraction, mass spectometric analyses (LC/MS with API-ES in positive modus) of the compounds showed a molecular mass attributed to the coupling of two molecules of the laccase substrate **1a** or **1b** with one molecule of **2c** accompanied by the loss of eight, six or four hydrogen atoms forming quinoid (**6a_1_**, **6b_1_**), mixed quinoid-hydroquinoid (**6a_2_**, **6b_2_**), and hydroquinoid trimers (**6a_3_**, **6b_3_**). ^1^H NMR analyses of the products also indicated heteromolecular trimers in each reaction. The hydroquinoid trimers (**6a_3_**, **6b_3_**) were the most prominent such structures determined.

### 3.4. Biological Activity of Biotransformation Products

The products **3a_1_** and **3b_1_** were those with the best supported structural data, and they were stable over a longer time period. Because of this, they were selected for assays of biological activity. The agar diffusion test was chosen for the determination of the antibacterial activity of the substances. Thereby, the size of the respective inhibition zone is a measure of the antibacterial efficacy.

Both of these products caused low to moderate growth inhibition of *S. aureus* and *S*. *epidermidis* strains, among them multidrug-resistant staphylococci (Table 4).

The educts **1a** and **2a** were not active against the strains tested, **1b** showed only very low activity with the highest concentration used. This indicates that products with antimicrobial activity can be produced by laccase-mediated reactions from two initially very poorly active or inactive compounds. 

HPLC measurements demonstrated good product stability for the compounds **3a_1_** and **3b_1_**, which had been stored in solid form at 4 °C for several weeks. However, incubation of the compounds **3a_1_** and **3b_1_** in aqueous solutions at 30 °C resulted in decomposition within some hours. For these reasons, the survey of their antimicrobial effects was restricted to the agar diffusion test.

**3a_1_** and **3b_1_** as well as **1a**, **1b**, and **2a** showed no cytotoxicity against FL cells at concentrations of 12.5, 25, 50, and 100 µg/mL. The growth of FL-cells was comparable with control culture (100–96% vitality).

## 4. Discussion

Because of increasing bacterial resistance to therapeutically used drugs, there is an urgent need to develop new antimicrobial agents. The laccase-mediated reaction of penicillin and cephalosporanic acid derivatives resulted in a dimerization by C−C or C−O bond formation and the synthesis of diastereomers, respectively [36,37]. These homomolecular products showed antimicrobial activity. Additionally, the laccase-mediated C−N bond formation between different partners resulted in heteromolecular products with antibacterial efficacy [48,49]. Such aminations are consequently a suitable green method for the synthesis of novel antibiotics. Thus, penicillins [38,40,41], cephalosporins [39,40,42], and its basic structures [46] have been structurally altered to achieve advanced antibacterial activities, especially against multidrug-resistant organisms. Furthermore, derivatives of the isolated antibacterial agents corollosporine from the marine fungus *Corollospora maritima* [50] and the ganomycins from *Ganoderma pfeifferi* [51] were transformed by one pot laccase-mediated reactions to improve their biological activity [38,42,52].

The sulfonamides and sulfones are antibacterial drugs that have been used for a long time [45,46], but whose value has progressively declined due to the emergence of resistant strains [53,54,55]. We have here derivatized antimicrobial sulfonamides and sulfones by laccase. Heterodimers and heterotrimers were synthesized by laccase-catalyzed reactions of sulfonamide antibiotics or dapsone with 2,5-dihydroxybenzene derivatives via nuclear amination. The formation of dimers (**3a**–**3f**, **5a**–**d**) was determined for all reactions of **2a**, **2b** with **1a**–**1e**. Trimers (**4f**, **6a**, **6b**) were only detected in the reactions of **2a**, **2b** with **1c** and for **2c** with **1a**, **1b**. The structure of these trimers differed depending on whether the starting compound possessed one (**2a**, **2b**) or two free amino groups (**2c**) accessible for a reaction with the *p*-hydroquinones (**1a**–**c**). Thus, the trimers formed with **2a** or **2b** consisted of one molecule **1c** and two molecules of **2a** or **2b**, respectively, whereas the trimers with **2c** were formed from two molecules of **1a** or **1b** and only one molecule of **2c**.

The dimers and trimers were detected in hydroquinoid or quinoid form, as described previously for heterodimers as products of *p*-hydroquinones with amino compounds such as aminopyrazole carboxamide [56] or aminothiophenol [21]. Interestingly, for the trimers with **2c,** also a mixed quinoid-hydroquinoid trimer was formed with one molecule of **1a** or **1b** as hydroquinone and one quinoid molecule of **1a** or **1b**.

The results regarding the biological activity showed that, with the exception of compound **1b**, the educts showed no inhibiting effect against the tested *Staphylococcus* species. Compound **1b** had low activity only at the highest concentration tested (1.27 µmol). In contrast, the products **3a_1_** and **3b_1_** were effective at concentrations between 0.14 to 0.74 µmol. This increase in antimicrobial activity of these products of laccase-catalyzed reactions is remarkable, given that previous attempts to use laccase-mediated transformation of β-lactam antibiotics (such as ampicillin, amoxicillin) did not lead to an increase in antibiotic efficacy [36,37,38].

The increased antibacterial efficacy for the products **3a_1_** and **3b_1_** in comparison to the educts as well as the inhibition of different *Staphylococcus* species, in particular the multidrug-resistant northern German epidemic MRSA, without toxicity for mammalian cells underlines the high potential of laccase-mediated reactions for the synthesis of novel antibiotics.

## Figures and Tables

**Table 1:** Heterodimers and heterotrimers produced by laccase-mediated reactions of 2,5-dihydroxybenzene derivatives (**1a**–**1c**) and sulfonamides (**2a**, **2b**).

Educts		Products			
		Quinoid Dimers	Hydroquinoid Dimers	Quinoid Trimers	Hydroquinoid Trimers
**1a** 8 R^1^=OCH_3_	**2a** R^2^=H	**3a_1_** 8 R^1^=OCH_3_ R^2^=H	nd ^1^	nd	nd
**1b** 8 9 10 R^1^=NHCH_2_CH_2_OH	**2a**	**3b_1_** 8 9 10 R^1^=NHCH_2_CH_2_OH R^2^=H	nd	nd	nd
**1c** 8 R^1^=CH_3_	**2a**	nd	**3c_2_** 8 R^1^=CH_3_ R^2^=H	**4c_1_** 8 R^1^=CH_3_ R^2^=H	nd
**1a** 8 R^1^=OCH_3_	**2b** R^2^= 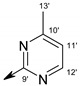	**3d_1_** 8 R^1^=OCH_3_ R^2^= 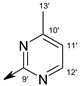	**3d_2_** 8 R^1^=OCH_3_ R^2^= 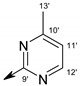	nd	nd
**1b** 8 9 10 R^1^=NHCH_2_CH_2_OH	**2b**	**3e_1_** 8 9 10 R^1^=NHCH_2_CH_2_OH R^2^= 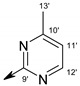	**3e_2_** 8 9 10 R^1^=NHCH_2_CH_2_OH R^2^= 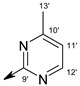	nd	nd
**1c** 8 R^1^=CH_3_	**2b**	**3f_1_** 8 R^1^=CH_3_ R^2^= 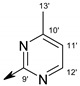	**3f_2_** 8 R^1^=CH_3_ R^2^= 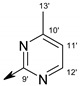	**4f_1_** 8 R^1^=CH_3_ R^2^= 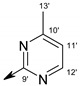	**4f_2_** 8 R^1^=CH_3_ R^2^= 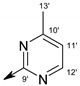

^1^ not detected by the measurements used.

**Table 2:** Heterodimers produced by laccase-mediated reactions of 2,5-dihydroxyphenylacetic acid (**1d**) and 2,5-dihydroxy-1,4-benzenediacetic acid (**1e**) and sulfonamides (**2a**, **2b**).

Educts		Products	
		Quinoid Dimers	Hydroquinoid Dimers
**1d** R^1^=H	**2a** R^2^=H	**5a_1_** R^1^=H R^2^=H	**5a_2_** R^1^=H R^2^=H
**1e** R^1^=CH_2_COOH	**2a**	**5b_1_** R^1^=CH_2_COOH R^2^=H	**5b_2_** R^1^=CH_2_COOH R^2^=H
**1d** R^1^=H	**2b** R^2^= 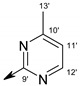	**5c_1_** R^1^=H R^2^= 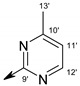	**5c_2_** R^1^=H R^2^= 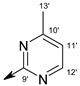
**1e** R^1^=CH_2_COOH	**2b**	**5d_1_** R^1^=CH_2_COOH R^2^= 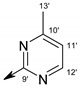	**5d_2_** R^1^=CH_2_COOH R^2^= 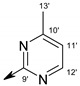

**Table 3:** Heterotrimers produced by laccase-mediated reactions of 2,5-dihydroxybenzene derivatives (**1a, 1b**) with dapsone (**2c**).

Educts		Products		
		Quinoid Trimers	Mixed Quinoid-Hydroquinoid Trimers	Hydroquinoid Trimers
**1a** 8 R^1^=OCH_3_	**2c**	**6a_1_** 8 R^1^=OCH_3_	**6a_2_** 8 R^1^=OCH_3_	**6a_3_** 8 R^1^=OCH_3_
**1b** 8 9 10 R^1^=NHCH_2_CH_2_OH	**2c**	**6b_1_** 8 9 10 R^1^=NHCH_2_CH_2_OH	**6b_2_** 8 9 10 R^1^=NHCH_2_CH_2_OH	**6b_3_** 8 9 10 R^1^=NHCH_2_CH_2_OH

**Table 4:** Antimicrobial activity of products **3a_1_** and **3b_1_**, educts **1a** to **1b** and **2a** against different *Staphylococcus* species; Zones of inhibition are given in diameter [mm] including paper disc (6 mm).

Substance	Amount *n* [µmol]	*S. aureus* ATCC6538	*S. aureus*-Northern German Epidemic MRSA	*S. epidermidis 99847*
**1a** (educt)	0.069	r *	r	r
	0.30	r	r	r
	0.74	r	r	r
	1.49	r	r	r
**1b** (educt)	0.069	r	r	r
	0.30	r	r	r
	0.74	r	r	r
	1.49	10	10	10
**2a** (educt)	0.058	r	r	r
	0.29	r	r	r
	0.73	r	r	r
	1.45	r	r	r
**3a_1_** (product of **1a** and **2a**)	0.030	r	r	r
	0.15	r	r	r
	0.37	r	12	r
	0.74	10	16	12
**3b_1_** (product of **1b** and **2a**)	0.030	r	r	r
	0.15	r	18	r
	0.37	20	22	20
	0.74	22	26	26

* resistant (no zone of inhibition).

## References

[1] Davies J., Davies D. (2010). Origins and evolution of antibiotic resistance. Microbiol. Mol. Biol. Rev..

[2] Ventola C.L. (2015). The antibiotic resistance crisis: Part 1: Causes and threats. Pharm. Ther..

[3] Guo Y., Song G., Sun M., Wang J., Wang Y. (2020). Prevalence and therapies of antibiotic-resistance in *Staphylococcus aureus*. Front. Cell Infect. Microbiol..

[4] Monti D., Ottolina G., Carrea G., Riva S. (2011). Redox reactions catalyzed by isolated enzymes. Chem. Rev..

[5] Sheldon R.A., Brady D., Bode M.L. (2020). The Hitchhiker’s guide to biocatalysis: Recent advances in the use of enzymes in organic synthesis. Chem. Sci..

[6] Witayakran S., Ragauskas A.J. (2009). Synthetic applications of laccase in green chemistry. Adv. Synth. Catal..

[7] Mogharabi M., Faramarzi M.A. (2014). Laccase and laccase-mediated systems in the synthesis of organic compounds. Adv. Synth. Catal..

[8] Romero-Guido C., Baez A., Torres E. (2018). Dioxygen activation by laccases: Green chemistry for fine chemical synthesis. Catalysts.

[9] Sousa A.C., Martins L.O., Robalo M.P. (2021). Laccases: Versatile biocatalysts for the synthesis of heterocyclic cores. Molecules.

[10] Leonowicz A., Edgehill R.U., Bollag J.-M. (1984). The effect of pH on the transformation of syringic and vanillic acids by the laccases of *Rhizoctonia praticola* and *Trametes versicolor*. Arch. Microbiol..

[11] Ciecholewski S., Hammer E., Manda K., Bose G., Nguyen V.T.H., Langer P., Schauer F. (2005). Laccase-catalyzed carbon-carbon bond formation: Oxidative dimerization of salicylic esters by air in aqueous solution. Tetrahedron.

[12] Mikolasch A., Schauer F. (2009). Fungal laccases as tools for the synthesis of new hybrid molecules and biomaterials. Appl. Microbiol. Biot..

[13] Bollag J.-M., Sjoblad R.D., Minard R.D. (1977). Polymerization of phenolic intermediates of pesticides by a fungal enzyme. Experientia.

[14] Jonas U., Hammer E., Haupt E.T.K., Schauer F. (2000). Characterisation of coupling products formed by biotransformation of biphenyl and diphenyl ether by the white rot fungus *Pycnoporus cinnabarinus*. Arch. Microbiol..

[15] Tatsumi K., Wada S., Ichikawa H., Liu S.Y., Bollag J.-M. (1992). Cross-coupling of a chloroaniline and phenolic-acids catalyzed by a fungal enzyme. Water Sci. Technol..

[16] Niedermeyer T.H.J., Mikolasch A., Lalk M. (2005). Nuclear amination catalyzed by fungal laccases: Reaction products of *p*-hydroquinones and primary aromatic amines. J. Org. Chem..

[17] Bollag J.-M., Liu S.-Y. (1985). Copolymerization of halogenated phenols and syringic acid. Pestic. Biochem. Phys..

[18] Benfield G., Bocks S.M., Bromley K., Brown B.R. (1964). Studies of fungal and plant laccases. Phytochemistry.

[19] Schlippert M., Mikolasch A., Hahn V., Schauer F. (2016). Enzymatic thiol Michael addition using laccases: Multiple C-S bond formation between *p*-hydroquinones and aromatic thiols. J. Mol. Catal. B-Enzym..

[20] Bhalerao U.T., Muralikrishna C., Rani B.R. (1994). Laccase enzyme-catalyzed efficient synthesis of 3-substituted-1,2,4-triazolo (4,3-b) (4,1,2) benzothiadiazine-8-ones. Tetrahedron.

[21] Hahn V., Mikolasch A., Weitemeyer J., Petters S., Davids T., Lalk M., Lackmann J.W., Schauer F. (2020). Ring-closure mechanisms mediated by laccase to synthesize phenothiazines, phenoxazines, and phenazines. ACS Omega.

[22] Anyanwutaku I.O., Petroski R.J., Rosazza J.P. (1994). Oxidative coupling of mithramycin and hydroquinone catalyzed by copper oxidases and benzoquinone. Implications for the mechanism of action of aureolic acid antibiotics. Bioorg. Med. Chem..

[23] Wellington K.W., Kolesnikova N.I. (2012). A laccase-catalysed one-pot synthesis of aminonaphthoquinones and their anticancer activity. Bioorg. Med. Chem..

[24] Zhang Y., Yao Q., Li Z., Yang F., Wang F., Liu J. (2019). A one-pot process for synthesis of mitomycin analogs catalyzed by laccase/lipase optimized by response surface methodology. Eng. Life Sci..

[25] Ünlü A.E., Prasad B., Anavekar K., Bubenheim P., Liese A. (2017). Investigation of a green process for the polymerization of catechin. Prep. Biochem. Biotech..

[26] Kurisawa M., Chung J.E., Uyama H., Kobayashi S. (2003). Laccase-catalyzed synthesis and antioxidant property of poly (catechin). Macromol. Biosci..

[27] Nicotra S., Cramarossa M.R., Mucci A., Pagnoni U.M., Riva S., Forti L. (2004). Biotransformation of resveratrol: Synthesis of *trans*-dehydrodimers catalyzed by laccases from *Myceliophtora thermophyla* and from *Trametes pubescens*. Tetrahedron.

[28] Lugaro G., Carrea G., Cremonesi P., Casellato M.M., Antonini E. (1973). Oxidation of steroid hormones by fungal laccase in emulsion of water and organic solvents. Arch. Biochem. Biophys..

[29] Nicotra S., Intra A., Ottolina G., Riva S., Danieli B. (2004). Laccase-mediated oxidation of the steroid hormone 17β-estradiol in organic solvents. Tetrahedron Asymmetry.

[30] Eggert C., Temp U., Dean J.F.D., Eriksson K.E.L. (1995). Laccase-mediated formation of the phenoxazinone derivative, cinnabarinic acid. FEBS Lett..

[31] Eggert C. (1997). Laccase-catalyzed formation of cinnabarinic acid is responsible for antibacterial activity of *Pycnoporus cinnabarinus*. Microbiol. Res..

[32] Osiadacz J., Al-Adhami A.J.H., Bajraszewska D., Fischer P., Peczynska-Czoch W. (1999). On the use of *Trametes versicolor* laccase for the conversion of 4-methyl-3-hydroxyanthranilic acid to actinocin chromophore. J. Biotechnol..

[33] Giurg M., Wiech E., Piekielska K., Gębala M., Młochowski J., Wolański M., Ditkowski B., Peczyńska-Czoch W. (2006). A new approach to synthesis of questiomycin A: Oxidative cyclocondensation of *ortho*-aminophenol. Pol. J. Chem..

[34] Giurg M., Piekielska K., Gębala M., Ditkowski B., Wolański M., Peczyńska-Czoch W., Młochowski J. (2007). Catalytic oxidative cyclocondensation of *o*-aminophenols to 2-amino-3H-phenoxazin-3-ones. Synth. Commun..

[35] Bruyneel F., Enaud E., Billottet L., Vanhulle S., Marchand-Brynaert J. (2008). Regioselective synthesis of 3-hydroxyorthanilic acid and its biotransformation into a novel phenoxazinone dye by use of laccase. Eur. J. Org. Chem..

[36] Agematu H., Tsuchida T., Kominato K., Shibamoto N., Yoshioka T., Nishida H., Okamoto R., Shin T., Murao S. (1993). Enzymatic dimerization of penicillin-X. J. Antibiot..

[37] Agematu H., Kominato K., Shibamoto N., Yoshioka T., Nishida H., Okamoto R., Shin T., Murao S. (1993). Transformation of 7-(4-hydroxyphenylacetamido) cephalosporanic acid into a new cephalosporin antibiotic, 7-[1-oxaspiro (2.5)octa-6-oxo-4,7-diene-2-carboxamido]cephalosporanic acid, by laccase. Biosci. Biotechnol. Biochem..

[38] Mikolasch A., Niedermeyer T.H.J., Lalk M., Witt S., Seefeldt S., Hammer E., Schauer F., Gesell M., Hessel S., Jülich W.D. (2006). Novel penicillins synthesized by biotransformation using laccase from *Trametes* spec. Chem. Pharm. Bull..

[39] Mikolasch A., Niedermeyer T.H.J., Lalk M., Witt S., Seefeldt S., Hammer E., Schauer F., Salazar M.G., Hessel S., Jülich W.D. (2007). Novel cephalosporins synthesized by amination of 2,5-dihydroxybenzoic acid derivatives using fungal laccases II. Chem. Pharm. Bull..

[40] Mikolasch A., Wurster M., Lalk M., Witt S., Seefeldt S., Hammer E., Schauer F., Jülich W.D., Lindequist U. (2008). Novel beta-lactam antibiotics synthesized by amination of catechols using fungal laccase. Chem. Pharm. Bull..

[41] Mikolasch A., Manda K., Schlüter R., Lalk M., Witt S., Seefeldt S., Hammer E., Schauer F., Jülich W.D., Lindequist U. (2012). Comparative analyses of laccase-catalyzed amination reactions for production of novel beta-lactam antibiotics. Biotechnol. Appl. Biochem..

[42] Mikolasch A., Hildebrandt O., Schlüter R., Hammer E., Witt S., Lindequist U. (2016). Targeted synthesis of novel beta-lactam antibiotics by laccase-catalyzed reaction of aromatic substrates selected by pre-testing for their antimicrobial and cytotoxic activity. Appl. Microbiol. Biotechnol..

[43] Domagk G. (1935). Chemotherapy of bacterial infections. Angew Chem.-Ger. Edit..

[44] Sköld O. (2000). Sulfonamide resistance: Mechanisms and trends. Drug Resist. Update.

[45] Wainwright M., Kristiansen J.E. (2011). On the 75th anniversary of Prontosil. Dyes Pigments.

[46] Zhu Y.I., Stiller M.J. (2001). Dapsone and sulfones in dermatology: Overview and update. J. Am. Acad. Dermatol..

[47] Witte W., Braulke C., Cuny C., Heuck D., Kresken M. (2001). Changing pattern of antibiotic resistance in methicillin-resistant *Staphylococcus aureus* from German hospitals. Infect. Cont. Hosp. Ep..

[48] Hahn V., Mikolasch A., Wende K., Bartrow H., Lindequist U., Schauer F. (2009). Synthesis of model morpholine derivatives with biological activities by laccase-catalysed reactions. Biotechnol. Appl. Biochem..

[49] Mikolasch A., Grunwald P. (2019). Laccase-mediated synthesis of novel antibiotics and amino acid derivatives. Pharmaceutical Biocatalysis: Chemoenzymatic Synthesis of Active Pharmaceutical Ingredients.

[50] Liberra K., Jansen R., Lindequist U. (1998). Corollosporine, a new phthalide derivative from the marine fungus *Corollospora maritima* Werderm. 1069. Pharmazie.

[51] Mothana R.A., Jansen R., Jülich W.D., Lindequist U. (2000). Ganomycins A and B, new antimicrobial farnesyl hydroquinones from the basidiomycete *Ganoderma pfeifferi*. J. Nat. Prod..

[52] Mikolasch A., Hessel S., Gesell Salazar M., Neumann H., Manda K., Gördes D., Schmidt E., Thurow K., Hammer E., Lindequist U. (2008). Synthesis of new *N*-analogous corollosporine derivatives with antibacterial activity by laccase-catalyzed amination. Chem. Pharm. Bull..

[53] Wise E.M., Abou-Donia M.M. (1975). Sulfonamide resistance mechanism in *Escherichia coli*-R plasmids can determine sulfonamide-resistant dihydropteroate synthases. Proc. Natl. Acad. Sci. USA.

[54] Vedantam G., Nichols B.P. (1998). Characterization of a mutationally altered dihydropteroate synthase contributing to sulfathiazole resistance in *Escherichia coli*. Microb. Drug Resist..

[55] Fiebelkorn K.R., Crawford S.A., Jorgensen J.H. (2005). Mutations in folP associated with elevated sulfonamide MICs for *Neisseria meningitidis* clinical isolates from five continents. Antimicrob. Agents Chemother..

[56] Hahn V., Davids T., Lalk M., Schauer F., Mikolasch A. (2010). Enzymatic cyclizations using laccases: Multiple bond formation between dihydroxybenzoic acid derivatives and aromatic amines. Green Chem..

